# Effects of fatigue on the activation characteristics and synergistic patterns of lower limb muscles during running

**DOI:** 10.3389/fphys.2026.1741432

**Published:** 2026-03-05

**Authors:** Zihao Li, Ke He

**Affiliations:** 1 Department of Track and Field, Institute of Physical Education and Training, Capital University of Physical Education and Sports, Beijing, China; 2 Sports Department, Beijing University of Aeronautics and Astronautics, Beijing, China

**Keywords:** exercise-induced fatigue, muscle co-activation, muscle synergy, running, surface electromyography

## Abstract

**Objective:**

This study aimed to investigate the effects of fatigue on the control mechanism of the lower limb neuromuscular system during running.

**Methods:**

The study participants were 25 male running enthusiasts (age: 20.9 ± 1.6 years; height: 174.8 ± 4.5 cm; body mass: 70.3 ± 5.2 kg) with more than 3 years of running experience. Surface electromyography (sEMG), motion capture, and heart rate monitoring technologies were used to obtain data for analysis in three dimensions: muscle activation, joint co-activation, and muscle synergy. The participants began running at 8 km/h, with the speed increased by 1 km/h every 2 min until their heart rate reached 75% of the estimated maximum heart rate (MHR), after which an individualized constant-speed phase was performed. Peak fatigue state was determined by two criteria: heart rate reaching 90% of MHR and a Borg scale score of ≥17. Data were compared before and after fatigue. Muscle synergies were extracted using non-negative matrix factorization (NNMF), and the number of modules was determined when the variance accounted for (VAF) first reached 95%.

**Results:**

The results showed that muscle activation exhibited phase-specific changes after fatigue: During the stance phase, the root mean square (RMS) values of the quadriceps femoris (QF) and gluteus maximus (GM) increased significantly (p < 0.05). During the swing phase, the RMS value of the tibialis anterior (TA) increased significantly while that of the GM decreased significantly (p < 0.05). The joint co-activation ratio (CAR) increased significantly only at the ankle joint during the swing phase (p < 0.05). There was no significant difference in the number of muscle synergy modules (p > 0.05). However, the activation timing of the early stance module was significantly advanced (p < 0.05), and the muscle weights within the modules also changed; for example, the weights of the vastus lateralis (VL) and lateral head of the gastrocnemius (GL) decreased, while the weight of the TA increased (p < 0.05).

**Conclusion:**

Our results indicate that under running-induced fatigue, the lower limb neuromuscular system maintains motor output based on the principle of “stability first” through three approaches: “selective compensation” to activate muscles, “rigidity enhancement” to stabilize distal joints, and “timing adjustment” to optimize synergy modules. The findings may contribute to training optimization and help reduce the likelihood of potential injuries.

## 1 Introduction

Running is a common and cost-effective form of aerobic exercise that significantly improves cardiorespiratory function and metabolic health while also enhancing lower limb muscle strength and neuromuscular coordination, thereby boosting exercise efficiency and physical stability (Akbar et al., 2022). However, long-term or high-intensity repetitive running subjects the lower limb muscles to sustained loads, easily inducing neuromuscular fatigue (Garrett et al., 2023). Muscle fatigue is a physiological state where muscle force output or power decreases during sustained or repetitive contractions, accompanied by impaired motor control. Its underlying mechanisms include metabolic disturbances and reduced ion channel function at the peripheral level, as well as altered central neural drive signals (Enoka and Duchateau, 2016). Fatigue during running not only reduces the efficiency of muscle contraction but also alters the motor control strategies of the central nervous system (CNS), thereby affecting gait stability and mechanical efficiency (Williams et al., 1991). Studies have shown that with the progression of fatigue, runners exhibit changes in stride length, stance phase duration, and joint kinematic parameters. Although such compensations can maintain motor output in the short term, prolonged fatigue may lead to overload of specific muscle groups and increased joint stress, thereby elevating the risk of overuse injuries (Yu et al., 2021).

At the individual muscle level, surface electromyography (sEMG) is widely used to evaluate the electrophysiological activity characteristics of muscles during running. The root mean square (RMS) value is a commonly used amplitude descriptor of rectified sEMG signals that reflects the overall intensity of muscle activation (De Luca, 1997). Existing studies have demonstrated that as running duration or exercise intensity increases, the RMS of major lower limb muscles, such as the rectus femoris (RF), VL, gastrocnemius, soleus, and tibialis anterior (TA), typically shows an upward trend. This indicates that the CNS maintains force output by increasing motor unit recruitment (Bonacci et al., 2009). However, in the deep fatigue phase, which in this study refers to a later stage of fatigue during prolonged or high-intensity running in which motor unit recruitment and firing strategies may be reorganized, RMS values may fluctuate or even decrease rather than increase monotonically, as suggested by the reports of previous EMG-based fatigue studies (Farina et al., 2014). One study found that during the fatigue phase of high-intensity running, the RMS of the quadriceps femoris (QF) and hamstrings (HS) decreased significantly, suggesting reduced motor neuron excitability and output efficiency (Burgess and Lambert, 2010). Different muscle groups also exhibit varying fatigue responses: the QF is more prone to decreased activation under fatigue, while the triceps surae shows minimal RMS changes, indicating stronger fatigue resistance (Jafarnezhadgero et al., 2025). Additionally, anterior and posterior muscle groups may exhibit compensatory activation patterns; when anterior muscles fatigue, posterior muscle activation increases to maintain gait stability and output efficiency (Paillard, 2012). However, most existing studies focus on activation changes in individual muscles, with limited research on the synergistic regulation mechanisms between muscles from the perspective of the entire lower limb muscle group, making it difficult to fully reveal the impact of fatigue on the overall control characteristics of the neuromuscular system.

Regarding joints, the co-activation relationship between antagonistic and synergistic muscles is a critical mechanism for maintaining joint stability and posture control. The co-activation ratio (CAR) is usually expressed as the ratio of RMS of muscle groups on both sides of a joint (Latash, 2023). For example, the RMS ratio of the QF to the hamstrings (HS) can be used to evaluate the dynamic coordination of the knee joint. Moderate co-activation helps increase joint stiffness, distribute impact forces, and maintain movement stability; however, excessive co-activation increases energy consumption and reduces exercise economy (Latash, 2018). Although hip joint co-activation is generally less dominant during steady-state running, it remains an integral component of the lower-limb kinetic chain and may indirectly influence neuromuscular coordination. During the progression of running fatigue, co-activation patterns typically exhibit biphasic changes: in the early phase, antagonistic muscle activation increases to maintain joint stability. In the deep fatigue phase, attenuation of central neural drive may be associated with decreased synergistic regulation capacity and reduced co-activation levels, manifesting as impaired joint control (Tam et al., 2017). For instance, one study reported a significant decrease in the quadriceps-hamstring CAR during the fatigue phase, accompanied by increased knee angular velocity fluctuations, indicating reduced coordination of the neuromuscular system (Zhang et al., 2021). Furthermore, the training level is considered a key factor influencing changes in co-activation. Experienced runners can maintain relatively stable co-activation levels under fatigue by optimizing activation timing and muscle group coordination to reduce mechanical energy loss, thereby demonstrating higher motor control stability (Möhler et al., 2022). However, current studies mostly focus on local joint muscle coordination and lack synergistic analysis between joints and muscle groups, failing to explain how the CNS redistributes drive strategies across different joints to maintain overall movement stability. Recent evidence indicates that fatigue-related changes in running performance and injury risk cannot be sufficiently explained by alterations in individual muscle activation alone but are closely associated with reorganization of intermuscular coordination and central neuromuscular control strategies. Fatigue adaptations have been shown to involve multilevel modulation across muscles and joints, reflecting higher-level neural regulation rather than isolated peripheral muscle fatigue. These findings highlight the limitations of single-level analyses and support the need for integrative approaches to investigate neuromuscular control under running-induced fatigue (Thomas et al., 2016; Yu et al., 2025).

Recently, the proposal of the muscle synergy theory has provided a new perspective for studying the adaptive mechanisms of motor control under fatigue. This theory posits that the CNS controls multiple muscle groups by activating a limited number of synergistic modules, thereby simplifying the complexity of motor control (Ting and Chvatal, 2010). These synergistic modules represent functionally linked muscle groups that are co-activated in a time-dependent manner to perform specific movement functions, such as support, propulsion, and cushioning. Healthy individuals typically exhibit 4–6 relatively stable muscle synergy modules during running (Nishida et al., 2017). However, under fatigue conditions, while the structure of these modules remains relatively stable, their activation weights and temporal distribution undergo significant changes, indicating that the CNS compensates for fatigue effects by adjusting the contribution ratio and time sequence of synergistic modules (Kakehata et al., 2024). One study found that under fatigue, the activation time of the propulsion module (dominated by the QF) was increased, while the activation of the support module (dominated by the gastrocnemius) was delayed (Hajilou et al., 2024). Nevertheless, this compensatory control often comes at the cost of reduced exercise economy, manifesting as lower energy utilization and weakened gait coordination (Saldanha et al., 2008). Although the synergy theory provides a new method for understanding the neural mechanisms of motor control, most existing studies have examined synergy structure or activation timing in isolation or have investigated muscle synergy adaptations without concurrent analysis of muscle activation and joint-level coordination. Consequently, how fatigue-induced adaptations at the muscle, joint, and synergy levels are coordinated within the same experimental framework remains unclear.

Using sEMG signals, this study aims to systematically analyze the changing patterns and internal connections of lower limb neuromuscular activity from the start of running to the fatigue phase, focusing on three aspects: the activation characteristics derived from sEMG signals and expressed using RMS values of individual lower limb muscles, the joint muscle CAR, and muscle synergy patterns. Through the combined analysis of multi-dimensional indicators, this study aims to reveal the comprehensive impact mechanism of fatigue on the neuromuscular control system during running, which may provide a scientific basis for training optimization, injury risk management, and research on motor control mechanisms. We hypothesized that running-induced fatigue would induce phase-specific changes in muscle activation and joint co-activation and alter the timing and weighting of muscle synergies while preserving the overall synergy structure.

## 2 Methods

### 2.1 Participants

Twenty-five male running enthusiasts (mean age of 20.92 ± 1.61 years, a height of 174.80 ± 4.54 cm, and a body mass of 70.32 ± 5.15 kg) were recruited from the student population of Capital University of Physical Education and Sports. The study’s inclusion criteria were as follows: 1) male participants with a heel-striking running pattern; 2) more than 3 years of regular running experience; and 3) a weekly running volume exceeding 30 km during the past 3 months. The exclusion criteria included any history of lower limb musculoskeletal injury, neurological disorders, or cardiovascular disease that could affect running performance. To avoid pre-existing fatigue, participants refrained from strenuous physical activity for at least 24 h prior to the experiment. All experimental testing sessions were conducted at a consistent time of day (between 14:00 and 17:00) to minimize potential circadian effects.

An *a priori* power analysis was conducted using G*Power software (version 3.1) for a paired-samples t-test with a two-tailed significance level of α = 0.05 and a desired statistical power of 0.80. Given the available sample size (n = 25), the minimum detectable effect size was estimated as Cohen’s d = 0.58, indicating that the present study was adequately powered to detect moderate-to-large within-subject effects, whereas smaller effects may not have been fully detectable. Additionally, sample sizes below 25 are commonly reported in surface EMG-based muscle synergy studies, largely due to the time-intensive experimental procedures and the high dimensionality of neuromuscular data processing. Furthermore, the sample size of the present study is comparable to those reported in previous muscle synergy investigations (Nishida et al., 2017; Kristiansen et al., 2016).

All participants provided written informed consent prior to participation. This study was approved by the Ethics Committee of Capital University of Physical Education and Sports (Approval No.: 2024A121).

### 2.2 Testing equipment

Three-dimensional motion capture system comprising an OptiTrack 8-camera motion capture system (NaturalPoint, United States) was used to collect spatial coordinates of anatomical landmarks at a sampling rate of 200 samples/s. The system’s Conventional Full Body 39-marker model was employed for marker placement.

Surface electromyography (sEMG) signals were acquired using a wireless sEMG system (Delsys Trigno, Delsys Inc., United States) at a sampling rate of 2000 Hz. Eight wireless dry surface electrodes were placed on the participants’ right lower limb muscles, including the rectus femoris (RF), vastus lateralis (VL), vastus medialis (VM), tibialis anterior (TA), gluteus maximus (GM), biceps femoris (BF), semitendinosus (ST), and lateral head of the gastrocnemius (GL). Electrode placement followed the recommendations of the SENIAM guidelines (Hajiloo et al., 2020), with electrodes positioned over the muscle belly and aligned with muscle fiber orientation. The inter-electrode distance was fixed according to the sensor configuration of the Trigno system, consistent with SENIAM placement principles, to minimize cross-talk and ensure signal reliability. The right lower limb was selected consistently across participants to improve data comparability.

**FIGURE 1:**
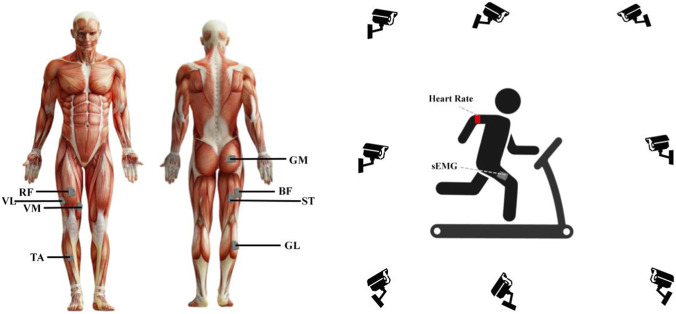
Experimental setup and sEMG electrode placement on the right lower limb. sEMG electrode placement on the right lower limb muscles: rectus femoris (RF), vastus lateralis (VL), vastus medialis (VM), tibialis anterior (TA), gluteus maximus (GM), biceps femoris (BF), semitendinosus (ST), and lateral head of gastrocnemius (GL). The schematic illustrations were created by the authors based on Delsys system-provided templates.

Regarding heart rate monitoring, a Polar H10 chest-worn heart rate sensor was used for continuous monitoring of participants’ real-time heart rate. The experiment was conducted on a Precor TRM833 treadmill (United States), with a running surface size of 153 × 51 cm (Figure 1).

### 2.3 Running fatigue test protocol

This study integrated physical functional fatigue and subjective perceived fatigue to quantify participants’ running fatigue level (Koblbauer et al., 2014; Colino et al., 2020). Participants started running at 8 km/h, with the speed increased by 1 km/h every 2 min. This gradual speed increase allowed participants to adapt to the exercise intensity, raise body and joint temperature, and activate physical functions until their heart rate reached 75% of the maximum heart rate (MHR), after which they entered the constant-speed running phase. Each participant’s MHR was estimated using the commonly applied formula: MHR = 220 − age (in years). The constant-speed running phase was performed at the final speed achieved at the end of the incremental stage.

During the constant-speed running phase, when a participant’s heart rate reached 90% of their MHR, their physical function was deemed to have reached the peak fatigue state. Additionally, participants were asked to rate their subjective perceived fatigue using the Borg scale every 2 min during constant-speed running. When a participant’s rating reached 17, their subjective perception was considered to have reached the peak fatigue state. When both physiological (heart rate) and subjective (RPE) fatigue criteria were satisfied, the participants continued running for an additional 1 min before the termination of the test. This additional running period was implemented to allow stabilization of the fatigued state prior to data collection, as neuromuscular adjustments and muscle synergy reorganization under fatigue are known to evolve progressively rather than occur instantaneously, and fatigue-related muscle synergy characteristics are recommended to be examined under a steady fatigued condition rather than at the onset of fatigue (Farina et al., 2014; Hug et al., 2010).

To eliminate the influence of running shoes on the participants’ running movements, all participants wore neutral cushioning running shoes (Nike Air Zoom Pegasus 34) provided by the laboratory.

### 2.4 Data collection and preprocessing

#### 2.4.1 Data collection timing

Within 1 min after participants entered the constant-speed running phase, data from 6 complete running cycles were collected and recorded as pre-fatigue data. After participants reached the peak fatigue state, data from another 6 complete running cycles were collected and recorded as post-fatigue data. Motion capture data and sEMG signals were synchronized using a hardware synchronization trigger. A common trigger signal simultaneously initiated data acquisition in both the motion capture system and the sEMG system, ensuring temporal alignment between the two datasets.

#### 2.4.2 Definition of a running cycle

A complete running cycle was defined as the time interval between one heel strike of the right foot and the next heel strike of the same foot (Dierks et al., 2010). Heel strike and toe-off events were determined based on motion capture data. Heel strike was identified when the vertical downward displacement of the heel marker in a single frame was less than 5 mm, whereas toe-off was identified when the vertical upward displacement of the forefoot marker exceeded 5 mm (Leitch et al., 2011). The <5 mm threshold was used solely as a gait event detection criterion rather than as a data filtering or exclusion condition.

#### 2.4.3 Phase division of running cycle

After referring to previous studies, a running cycle was further divided into two main phases (Lohman et al., 2011). The segmentation of the running cycle and its sub-phases was based on gait events identified from synchronized motion capture data. (1) Stance phase:The stance phase extended from heel strike of the supporting leg to toe-off of the same leg. This was further subdivided into the early stance phase (first 50% of the stance phase) and late stance phase (last 50% of the stance phase). (2) Swing phase:The stance phase extended from toe-off of the supporting leg to the next heel strike of the same leg. This was further subdivided into the early swing phase (from the start of the swing phase to 50% of the swing phase), mid-swing phase (50%–85% of the swing phase), and late swing phase (last 15% of the swing phase).

#### 2.4.4 sEMG signal preprocessing

The sEMG signals segmented by running cycle were filtered using a 4th-order Butterworth band-pass filter (cutoff frequencies: 10–500 Hz). The filtered signals were full-wave rectified. A 4th-order Butterworth low-pass filter (cutoff frequency: 10 Hz) was applied to the rectified signals to obtain the sEMG signal envelope. The amplitude of the sEMG envelope from each channel was normalized to its maximum value within the analyzed trials. Subsequently, RMS values were calculated from the processed sEMG signals using a moving window and were used as an amplitude descriptor to quantify muscle activation intensity for subsequent analyses. The normalized signals were resampled to cover 0%–100% of the running cycle (Saito et al., 2018). The sEMG curves from six running cycles of each participant were averaged.

### 2.5 Analysis indicators

The analysis indicators included sEMG root mean square (RMS) value, muscle CAR, and muscle synergy analysis.

#### 2.5.1 Root mean square

The Root Mean Square (RMS) was calculated for the QF, TA, GM, HS, and GL. The RMS value of QF was the sum of the RMS of RF, VL, and VM. The RMS value of HS was the sum of the RMS of the ST and BF.

#### 2.5.2 Muscle Co-Activation ratio

Muscle Co-Activation Ratio (CAR) were calculated for the knee joint and ankle joint, based on RMS using the formula:

CAR=RMSofantagonistmuscles/RMSofagonistmuscles

For the knee joint, the HS served as the antagonist muscle, and the QF served as the agonist muscle. For the ankle joint, the TA served as the antagonist muscle, while the GL served as the agonist muscle.

#### 2.5.3 Muscle synergy analysis

The analysis of muscle synergy included three aspects: the number of synergy modules, the peak timing of the activation coefficient of each module, and the main synergistic muscles (MSMs) in each module.

The classical Gaussian non-negative matrix factorization (NNMF) algorithm was used to extract muscle synergies. In this method, the muscle activation model represents the sEMG signals, which can be decomposed into two matrices. One of them is the muscle synergy vector matrix (W, relative weights of muscles within each module), and the other is the time-varying activation coefficient (C). The decomposition model can be expressed using the following formula:

$$V_{\text{mn}}\approx {\left(\text{WC}\right)}_{\text{mn}}=\sum_{i=1}^{k}W_{\text{mi}}C_{\text{in}}=V_{\text{mn}}^{\prime }$$

where $V_{\text{mn}}$ represents *m* channels of sEMG signals with n sampling points, *k* represents the number of synergy modules, and *W* reflects the activation weight of each muscle in the *i*th synergy module. In this study, the W value was normalized to 0–1. When the weight values of these muscles are above 0.3, they are considered as main synergistic muscles (MSMs) (Kim et al., 2018). C is the time-varying activation coefficient, which represents the contribution of the *i*th synergy matrices to the movement at time *t*. $V_{\text{mn}}^{\prime }$ represents the reconstructed sEMG signals.

The variance accounted for (VAF) was used to identify the optimal number of synergy modules k. The VAF can be expressed as follows:

$$\text{VAF}=1-\frac{\text{RSS}}{\text{TSS}}=1-\frac{\sum {\left(V_{\text{mn}}-V_{\text{mn}}^{\prime }\right)}^{2}}{\sum {\left(V_{\text{mn}}\right)}^{2}}$$

where RSS refers to the residual sum of squares and TSS refers to the total sum of squares. The number of synergies was determined such that the VAF was at least 95%.

The Pearson correlation coefficient was used to assess the similarity of the decomposed muscle synergy vector matrices at different fatigue stages. When the correlation coefficient (r) was greater than 0.6, the vectors were classified as the same type of muscle synergy and merged accordingly (Saito et al., 2018).

### 2.6 Statistical analysis

Statistical analyses were performed using SPSS version 26.0 (IBM Corp., Armonk, NY, United States). The Shapiro–Wilk test was used to assess the normality of all outcome variables. The results indicated that the RMS values of individual muscles, muscle CAR, the number of muscle synergy modules, the peak timing of activation coefficients, and the weighting of MSMs were approximately normally distributed. Therefore, parametric statistical tests were applied for these variables.

Paired-samples t-tests were used to compare pre- and post-fatigue differences in muscle RMS values, muscle CARs, and the number of synergy modules. For muscle synergy analyses, independent-samples t-tests were used to compare the peak timing of activation coefficients and the weighting of MSMs between pre- and post-fatigue conditions after module classification. Effect sizes for significant results were quantified using Cohen’s d. Statistical significance was set at p < 0.05.

## 3 Results

### 3.1 Muscle RMS

**FIGURE 2:**
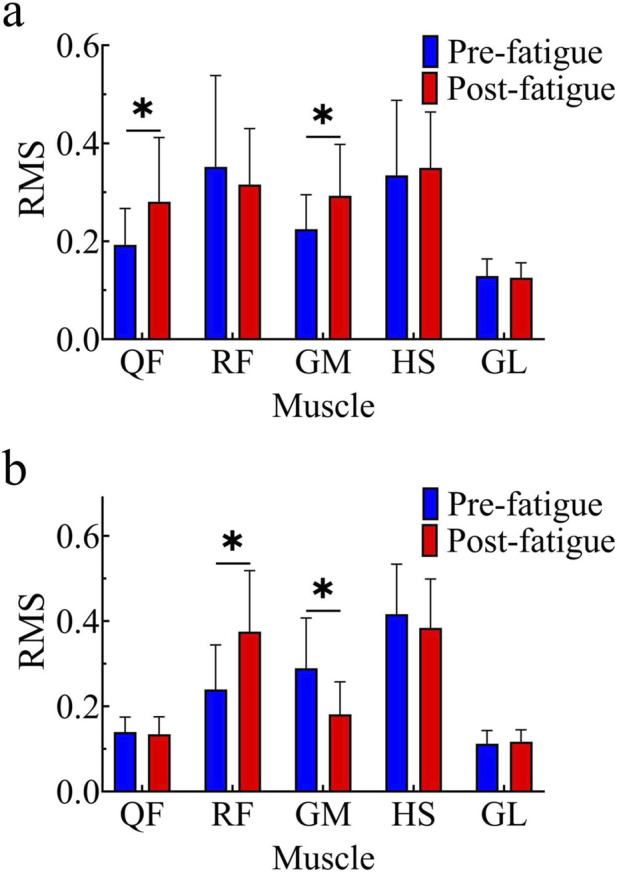
Comparison of muscle RMS values before and after fatigue; **(a)** Stance phase; **(b)** Swing phase; *, p < 0.05.

The RMS values of each muscle during the stance phase are shown in Figure 2a. Among them, the RMS values of the QF and GM after fatigue were significantly higher than those before fatigue (QF: t = −2.94, p < 0.05, 95% CI [−0.150, −0.026], Cohen’s d = 0.59; GM: t = −2.59, p < 0.05, 95% CI [−0.122, −0.014], Cohen’s d = 0.52).

The RMS values of each muscle during the swing phase are shown in Figure 2b. The RMS value of the TA after fatigue was significantly higher than that before fatigue (t = −3.75, p < 0.05, 95% CI [−0.211, −0.061], Cohen’s d = 0.75), whereas the RMS value of the GM after fatigue was significantly lower than that before fatigue (t = 4.46, p < 0.05, 95% CI [0.058, 0.158], Cohen’s d = 0.89).

### 3.2 Muscle CAR

**FIGURE 3:**
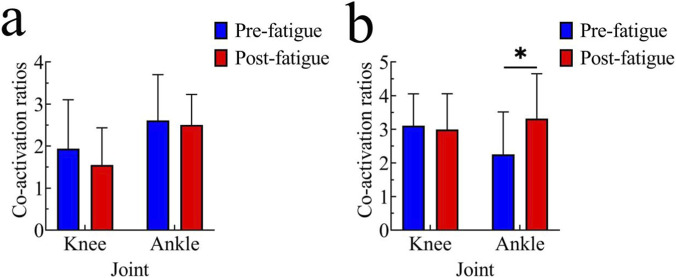
Comparison of muscle co-contraction ratios before and after fatigue; **(a)** Stance phase; **(b)** Swing phase; *, p < 0.05.

The muscle CARs during the stance phase are shown in Figure 3a. No significant differences were observed in the CARs of the knee and ankle joints before and after fatigue.

The muscle CARs during the swing phase are shown in Figure 3b. There was no significant difference in the CAR of the knee joint before and after fatigue; however, the CAR of the ankle joint after fatigue was significantly higher than that before fatigue (pre-fatigue: 2.25 ± 1.26; post-fatigue: 3.32 ± 1.33; t = −2.87, p < 0.05, 95% CI [−1.826, −0.299], Cohen’s d = 0.57).

### 3.3 Muscle synergies

#### 3.3.1 Number of synergy modules

**FIGURE 4:**
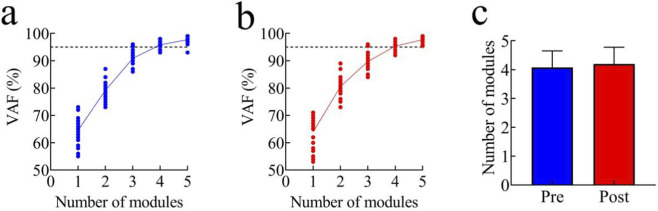
Number of synergy modules; **(a)** VAF curve before fatigue; **(b)** VAF curve after fatigue; **(c)** Comparison of the number of modules before and after fatigue.

The number of synergy modules before and after fatigue is presented in Figure 4. When the VAF first reached 95%, three participants exhibited three synergy modules, 17 participants exhibited four modules, and five participants exhibited five modules before fatigue. After fatigue, two participants exhibited three modules, 16 participants exhibited four modules, and seven participants exhibited five modules. No significant difference was observed in the number of synergy modules between the pre-fatigue and post-fatigue conditions (pre-fatigue: 4.08 ± 0.57; post-fatigue: 4.20 ± 0.58; t = −0.901, p > 0.05, 95% CI [−0.394, 1.548], Cohen’s d = 0.80).

#### 3.3.2 Peak timing of activation coefficients

**FIGURE 5:**
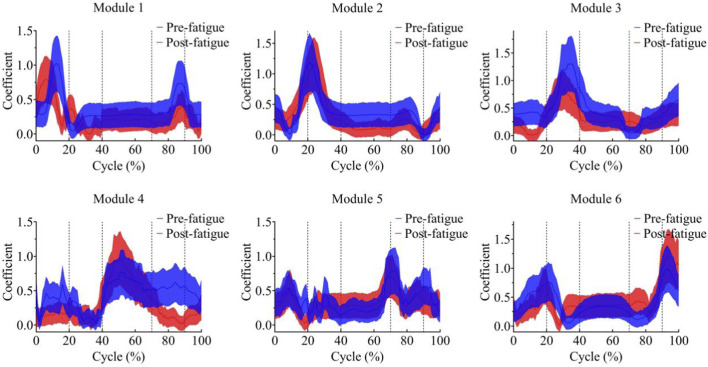
Activation curves of each module before and after fatigue; Shaded areas represent standard deviation.

Pearson correlation analysis was performed on the synergy modules before and after fatigue. Modules with correlation coefficients greater than 0.60 were merged to classify synergy types, resulting in six distinct synergy modules. The activation curves of the six modules are shown in Figure 5.

In Module 1, the peak of the activation coefficient occurred at 13.4% ± 2.3% of the gait cycle before fatigue and at 6.8% ± 2.8% after fatigue; this module primarily controlled the early stance phase of running. In Module 2, the activation coefficient peaked at 22.1% ± 3.1% before fatigue and at 24.5% ± 2.8% after fatigue, corresponding to lower-limb control during the early stance phase, with the peak occurring slightly later than in Module 1. The activation coefficient for Module 3 peaked at 35.8% ± 5.5% before fatigue and at 35.4% ± 4.4% after fatigue, representing the late stance phase. Module 4 exhibited peak activation at 52.0% ± 4.7% before and 52.0% ± 3.7% after fatigue, corresponding to the early swing phase. Module 5 peaked at 73.7% ± 2.2% before fatigue and 71.7% ± 4.7% after fatigue, corresponding to the mid-swing phase, while Module 6 peaked at 94.2% ± 3.7% before fatigue and 95.5% ± 2.4% after fatigue, representing the late swing phase.

**FIGURE 6:**
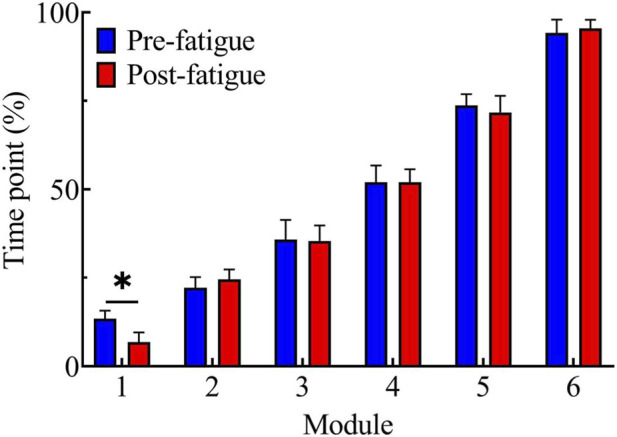
Comparison of activation peak timing of each module before and after fatigue; *, p < 0.05.

Independent-samples t-tests revealed that the peak activation timing of Module 1 was significantly earlier in the post-fatigue condition compared with the pre-fatigue condition, t = 9.40, p < 0.05, 95% CI [5.01, 8.19] (Figure 6).

#### 3.3.3 Main synergistic muscles

**FIGURE 7:**
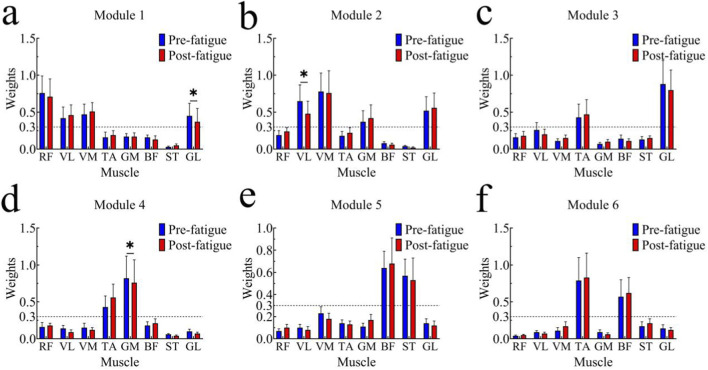
Comparison of main synergistic muscles in each module before and after fatigue; *, p < 0.05. The subfigures **(a-e)** correspond to synergistic modules 1-6.

The main synergistic muscles (MSMs) of each module are shown in Figure 7. Muscles with contribution weightings greater than 0.3 were identified as the main contributors within each synergy module.

Module 1 was mainly composed of the quadriceps muscles (RF, VL, and VM) together with the GL. Module 2 primarily included the VL, VM, GM, and GL as major contributors. In Module 3, the dominant contributors were the TA and GL, whereas Module 4 was mainly characterized by contributions from the TA and GM. Modules 5 and 6 were predominantly composed of hamstring-related muscles, with the BF and ST showing the highest contribution weightings.

Independent-samples t-tests revealed several significant differences between the pre-fatigue and post-fatigue conditions. In Module 1, the weighting of the GL was significantly lower after fatigue (p < 0.05, t = 2.881, 95% CI = [0.025, 0.159], Cohen’s d = 0.45). In Module 2, the weighting of the VL was significantly reduced following fatigue (p < 0.05, t = 4.843, 95% CI = [0.123, 0.310], Cohen’s d = 0.75). In Module 4, the weighting of the TA was significantly higher after fatigue (p < 0.05, t = −3.327, 95% CI = [−0.230, −0.052], Cohen’s d = 0.55).

## 4 Discussion

This study investigated male recreational runners with more than 3 years of running experience and a weekly mileage exceeding 30 km in the past 3 months. Using a pre- and post-fatigue comparison design combined with wireless surface electromyography (sEMG), motion capture, and heart rate monitoring, this study systematically analyzed changes in lower limb muscle RMS values, joint CAR, and muscle synergy patterns under fatigue. The objective was to comprehensively elucidate the neuromuscular regulation mechanisms of lower-limb function during running-induced fatigue.

### 4.1 Effects of fatigue on lower-limb muscle RMS and physiological mechanisms

Our results showed that muscle RMS values exhibited phase-specific changes after fatigue. During the stance phase, the RMS amplitudes of the QF and GM were significantly increased. During the swing phase, the RMS of the TA increased significantly, while that of the GM decreased. These differentiated responses reflect adaptive neural regulation strategies that are closely associated with functional demands and the training background of the participants.

Functionally, in the stance phase, the QF serves as a primary extensor, providing knee stabilization and shock absorption. The increased RMS after fatigue indicates a central compensatory mechanism; when muscle force output declines, the CNS recruits additional motor units or increases their firing rates to maintain required joint stiffness and impact attenuation (Semmler et al., 2007). This finding aligns with the reports of previous studies showing enhanced VL activation following fatigue (Giandolini et al., 2016). The elevated activation of the GM during the stance phase relates to hip stabilization demands: as trunk stability decreases under fatigue, the CNS reinforces the activity of the GM to control hip extension range and prevent excessive forward lean (Zandbergen et al., 2023). Conversely, reduced activation of the GM during the swing phase may represent an energy-saving “functional trade-off” strategy, as by suppressing nonessential activation, the CNS conserves metabolic resources to sustain prolonged running (Ivanenko and Gurfinkel, 2018).

The enhanced activation of TA during the swing phase has clear functional significance. As the primary ankle dorsiflexor, TA prevents foot drop and prepares for initial contact. Its increased activation following fatigue indicates anticipatory postural control for ground impact. This finding is consistent with those of previous studies showing elevated pre-activation of the TA under fatigue, reinforcing its role in protective foot posture control (Coventry et al., 2006). In contrast, the stable activation of the HS across phases differs from reports of fatigue-induced HS reduction in untrained individuals. The discrepancy likely arises from better fatigue resistance in trained runners due to improved oxidative metabolism and endurance capacity, which allowed the HS to maintain stable activation even at high perceived exertion (Fiorenza et al., 2019). This highlights the positive regulatory effect of endurance training on muscle fatigue adaptation.

Overall, fatigue did not cause a generalized decline in muscle activation but rather a differentiated pattern of enhancement and reduction across specific muscle groups. This selective compensation strategy allows short-term maintenance of movement stability; however, prolonged asymmetrical loading may accelerate localized overuse, increasing injury risk (Van Der Horst et al., 2015). These findings expand upon previous EMG-based fatigue studies by demonstrating that trained runners exhibit phase-specific muscle activation adjustments rather than a uniform decline in activation intensity.

### 4.2 Effects of fatigue on joint CAR and implications for injury risk

Changes in joint CAR directly reflect the coordination between agonist and antagonist muscles and are critical indicators of joint stability and injury risk. Our results revealed distinct phase-dependent CAR responses to fatigue. During the stance phase, both knee and ankle CARs showed no significant changes. During the swing phase, the ankle CAR significantly increased, while the knee CAR remained unchanged. This finding suggests that the CNS adopts differentiated control strategies for different joints and gait phases when fatigue develops.

The absence of CAR changes during the stance phase may be related to the participants’ long-term training background, which likely enhanced their neuromuscular coordination (Petersen et al., 2008). Even under fatigue, the CNS maintained the necessary joint stability for ground contact: coordinated activation of the QF and HS provided knee control and impact absorption, while balance between the TA and gastrocnemius ensured proper ankle positioning (Komi, 2000). This differs from the results observed in untrained individuals, indicating that consistent training strengthens central control of stance-related synergies and reduces instability under fatigue (Santos, 2024).

In contrast, the increased ankle CAR during the swing phase appears to result from higher TA activation combined with relatively stable GL activation. This suggests that the CNS prioritized distal joint stiffness to preserve stability and force transmission. The ankle joint, as a key site for ground interaction and energy storage, is crucial for both balance and propulsion. Enhanced co-activation of the GL and TA likely increases ankle stiffness to attenuate vertical vibration and limit excessive mediolateral rolling, mitigating the risk of missteps or structural deviations (Teruya et al., 2023). However, this also implies greater energy expenditure and mechanical resistance, potentially reducing running economy during prolonged or severe fatigue. The lack of knee CAR change again indicates preserved dynamic coordination between QF and HS, suggesting that proximal control mechanisms remain robust in moderate fatigue. Overall, our results demonstrate a hierarchical regulatory strategy: proximal joints (e.g., knee) emphasize coordinated balance, while distal joints (e.g., ankle) increase stiffness to offset instability. This adaptive shift from “flexible control” to “rigid stabilization” reflects the CNS’s prioritization of stability under fatigue. This interpretation is supported by recent findings showing that fatigue-related changes in running performance and injury risk are more strongly associated with alterations in intermuscular and inter-joint coordination than with isolated muscle activation deficits (Yu et al., 2025).

### 4.3 Fatigue-induced reorganization of muscle synergy patterns and central control logic

According to the muscle synergy theory, the CNS achieves complex motor control through a limited set of coordinated modules. Therefore, fatigue provides direct insight into central regulatory strategies. In this study, NNMF was used to extract synergy modules. Our results showed no significant difference in the number of synergy modules before and after fatigue; however, the timing of peak activation and the weighting of key muscles changed significantly. These changes corresponded closely to functional demands of the gait cycle, revealing a central adaptation principle characterized by “structural conservation with functional modulation.”

The average number of modules at 95% of VAF was 4.08 ± 0.57 pre-fatigue and 4.20 ± 0.58 post-fatigue, with four modules most frequently observed (17 pre-fatigue, 16 post-fatigue). This finding supports the reports of prior research indicating that fatigue does not alter synergy structure (Wang et al., 2025). The stability of the synergy number suggests that the CNS retains core coordination modules to maintain the integrity of movement patterns. Maintaining this modular structure contributes to movement economy, as no additional modules are required to compensate for fatigue, minimizing redundant control and energy cost, which is consistent with the efficient locomotor patterns developed through long-term training (Mileti et al., 2020). Similar conclusions have also been reported in recent neuromuscular control studies, which demonstrated that fatigue predominantly affects the temporal activation patterns and muscle weightings within existing synergies, while the overall modular structure remains preserved (Chang and Hu, 2025).

However, significant temporal and structural adjustments occurred within the modules. The activation peak of the early-stance module advanced notably, while muscle contributions shifted—particularly a decrease in VL and GL weightings and an increase in TA weighting. This implies that the CNS reconfigures intra-module composition and activation timing to sustain movement stability (Park and Caldwell, 2022). The earlier activation of extensor-dominant modules enhances ground-reaction absorption during early stance, while increased TA weighting strengthens swing-phase control, compensating for reduced plantar-flexor output. Therefore, fatigue adaptation occurs not by recruiting new muscles but by reorganizing temporal and structural coordination within existing modules. These results align with those of prior research showing that fatigue primarily induces functional reorganization rather than structural disruption of synergy control (Kakehata et al., 2024). The simultaneous presence of stable module quantity and flexible activation timing demonstrates a dual control property of “structural rigidity and temporal flexibility (Fan et al., 2024).” This underscores the capacity of synergy analysis to reveal high-level neural control mechanisms beyond what individual sEMG or CAR metrics can capture.

Collectively, the findings of this study confirm that the lower-limb neuromuscular system undergoes multilevel functional reorganization during running-induced fatigue. At the muscle level, activation patterns diverge across groups, reflecting selective compensation. At the joint level, distal control becomes stiffer, highlighting hierarchical adaptation. At the synergy level, activation timing and weighting redistribute to preserve dynamic stability. The unchanged number of synergy modules suggests that the overall modular structure of neuromuscular control is preserved under fatigue, whereas significant changes occur in muscle weightings and activation timing within specific modules. The combined analysis of RMS, CAR, and synergy characteristics allows a more complete interpretation of fatigue-related neuromuscular adjustments than approaches based on a single variable.

### 4.4 Practical implications

From a practical perspective, the present findings suggest that fatigue-resistant running performance relies more on the redistribution of muscle activation and coordination rather than changes in overall control structure. Therefore, training programs aiming to enhance fatigue tolerance may benefit from emphasizing phase-specific neuromuscular control and distal joint stability, rather than focusing solely on increasing muscle strength. Additionally, monitoring changes in muscle weighting and activation timing may provide sensitive indicators for early detection of maladaptive fatigue responses and potential injury risk.

### 4.5 Limitation

This study has some limitations. Joint-level co-activation analysis was limited to the knee and ankle joints, and hip joint co-activation was not included as a primary outcome due to methodological constraints associated with non-invasive surface electromyography and the comparatively lower emphasis on hip co-activation in running biomechanics. Consequently, direct comparison with studies focusing on proximal joint control and injury mechanisms is partially restricted. Although exploratory analyses using available hip-related muscles did not reveal significant pre- and post-fatigue differences, hip joint co-activation was not retained as a main outcome variable because of interpretative uncertainty and signal acquisition limitations. Furthermore, the investigation targeted acute running-induced fatigue in trained recreational runners, which may limit the generalizability of the findings to other populations with different training backgrounds, fatigue resistance, or neuromuscular control strategies. Although similar analytical approaches have been reported in previous research, the present study integrates muscle activation intensity, joint-level coordination, and muscle synergy organization within a unified framework. Nevertheless, the cross-sectional design cannot fully address long-term neuromuscular adaptations or cumulative fatigue effects. Future studies should expand joint-level coordination analyses to the hip, explore different fatigue magnitudes and durations, and apply longitudinal designs to better elucidate hierarchical neuromuscular regulation under running-induced fatigue.

## 5 Conclusion

With running-induced fatigue, the RMS values of the QF and GM increased during the stance phase, while the activity of the TA rose and that of the GM decreased during the swing phase, reflecting a compensatory strategy by the CNS to sustain function. The ankle CAR increased significantly only during the swing phase, indicating that the CNS enhanced ankle stiffness while maintaining knee coordination for stability. Although the number of synergy modules remained unchanged, the activation peak of the early stance module shifted earlier, with reduced GL weighting in Module 1, decreased VL weighting in Module 2, and increased TA weighting in Module 4. These findings show that the CNS adapts to fatigue primarily through adjustments in synergy timing and muscle weighting rather than structural changes. Overall, the CNS prioritizes stability through a multilevel strategy involving muscle activation, joint regulation, and muscle synergy reorganization, supporting fatigue management, injury prevention, and individualized endurance training programs.

## Data Availability

The raw data supporting the conclusions of this article will be made available by the authors, without undue reservation.
